# Current Status of Synthetic Mammalian Embryo Models

**DOI:** 10.3390/ijms252312862

**Published:** 2024-11-29

**Authors:** Haneul Kim, Eunhye Kim

**Affiliations:** Laboratory of Molecular Diagnostics and Cell Biology, College of Veterinary Medicine, Gyeongsang National University, Jinju 52828, Republic of Korea; skyk@gnu.ac.kr

**Keywords:** embryogenesis, synthetic embryo model, blastoid, gastruloid, 3D stem cell culture technology

## Abstract

Advances in three-dimensional culture technologies have facilitated the development of synthetic embryo models, such as blastoids, through the co-culturing of diverse stem cell types. These in vitro models enable precise investigation of developmental processes, including gastrulation, neurulation, and lineage specification, thereby advancing our understanding of early embryogenesis. By providing controllable, ethically viable platforms, they help circumvent the limitations of in vivo mammalian embryo studies and contribute to developing regenerative medicine strategies. Nonetheless, ethical challenges, particularly regarding human applications, persist. Comparative studies across various species—such as mice, humans, non-human primates, and ungulates, like pigs and cattle—offer crucial insights into both species-specific and conserved developmental mechanisms. In this review, we outline the species-specific differences in embryonic development and discuss recent advancements in stem cell and synthetic embryo models. Specifically, we focus on the latest stem cell research involving ungulates, such as pigs and cattle, and provide a comprehensive overview of the improvements in synthetic embryo technology. These insights contribute to our understanding of species-specific developmental biology, help improve model efficiency, and guide the development of new models.

## 1. Introduction

Advancements in three-dimensional (3D) culture technologies have facilitated the development of blastoid and synthetic embryo models through the co-culturing of various types of stem cells [1,2]. Given that mammalian embryos are located within the uterus and cannot be observed directly or manipulated, conducting in vivo research on the development of these embryos is highly challenging [3]. Accordingly, the development of in vitro models of peri- and post-implantation embryos is crucial for studying the mechanisms underlying cell-to-cell interactions and morphogenesis during early embryogenesis, including key processes, such as gastrulation, neurulation, and the initial formation of major organs. Research in this context has considerable implications for the advancement of regenerative medicine, as an understanding of these early developmental stages can inform strategies for tissue engineering and the treatment of developmental disorders.

However, research on human embryos is fraught with ethical and regulatory challenges. Culturing embryos beyond 14 days is prohibited, and growing synthetic embryos in endometrial organoids is restricted [4]. Consequently, alternative models, such as the gastruloid, which cannot develop into a fetus but allow for the study of gastrulation, are gaining attention [5,6,7]. These models provide a valuable platform for investigating early human developmental events without breaching ethical boundaries.

For several decades, rodents have been extensively used as model organisms for mammalian studies owing to their cost-effectiveness, short gestation periods, and genetic tractability. Nonetheless, rodents do not accurately reflect human embryonic development mechanisms due to significant physiological and anatomical differences. These differences necessitate the use of alternative models to better understand human-specific developmental processes. Despite this need, research on non-rodent models is relatively limited.

In this review, we summarize the recent advancements in embryo developmental research across different species, focusing on mice, humans, and non-human primates. Additionally, by examining the current status of stem cell research around ungulates, including pigs, we aim to provide new insights into strategies that could be used to develop more accurate embryonic models. This comprehensive approach seeks to bridge the gap between animal models and human embryogenesis, ultimately contributing to a more nuanced understanding of mammalian development and its applications in regenerative medicine.

## 2. Comparison of Embryogenesis in Mice and Other Mammals

The periods and trends of embryonic development vary considerably across mammalian species, including mice, humans, monkeys, cows, and pigs. The totipotent state, which allows a single cell to produce both embryonic and extraembryonic lineages, is maintained from the zygote stage up to the two- to four-cell stage in mice [8] and from the four- to eight-cell stage in humans [9], cows [10], pigs [11], and other mammals [12,13]. This totipotent phase sets the foundation for subsequent cellular differentiation and the transition to a more specialized pluripotent state. As the zygote undergoes successive cell divisions, it initially relies on maternal RNA and proteins deposited in the oocyte to guide early developmental processes. However, this maternal contribution is finite and must eventually be replaced by the embryo’s own genetic program. This transition is marked by embryonic genome activation (EGA), a crucial process where the control of development shifts from maternal RNA to the embryonic genome, initiating the expression of the embryo’s own genes [14]. EGA occurs at different stages across species: in mice, EGA begins at the two-cell stage; in humans, monkeys, and cows, it is initiated at the four- to eight-cell stage; and in pigs, it starts at the four-cell stage [14,15]. This activation is crucial for establishing the transcriptional program necessary for the embryo’s early development and differentiation, occurring just before the compaction stage.

These differences highlight the diversity in developmental timelines and mechanisms among various species, underscoring the importance of comparative studies to unravel the unique and conserved aspects of mammalian embryogenesis.

### 2.1. Compaction Mechanisms

In mammalian embryogenesis, compaction is an essential event that occurs before the blastocyst is formed (Table 1).

During compaction, cell-to-cell adhesion increases, intercellular contact is maximized, and a tightly packed mass of cells, known as the morula, is formed. This process is crucial for the successful development of the embryo and is conserved across all mammalian species, although the timing of its initiation and completion varies [16,17]. In mice, compaction begins at the eight-cell stage [18], whereas in humans, it can start as early as the four-cell stage and typically occurs around the eight-cell stage, although it can also extend up to the sixteen-cell stage [19]. By contrast, rhesus and baboon monkeys [20,21] exhibit less pronounced compaction, whereas in cattle [22], compaction occurs at the 16–32-cell stage. In pigs, compaction is not fully established until just before blastocyst formation [23]. Despite its fundamental role, the mechanisms driving compaction in humans and other mammalian species, excluding mice, remain poorly understood. In mouse embryos, this process is driven and facilitated by the reorganization of the actomyosin cytoskeleton and the upregulation of cell adhesion molecules such as E-cadherin [24]. E-cadherin plays a critical role in mediating cell–cell adhesion, thereby ensuring the proper alignment and integration of cells within the developing embryo. E-cadherin dysfunction leads to the failure of compaction, underscoring its essential role in this process [25]. Understanding these compaction mechanisms in detail could provide crucial insights into early embryonic development and the potential causes of developmental disorders in mammals.

### 2.2. Polarization and Signaling Pathways Involved in Cell Fate Decisions

As compaction progresses, cells undergo polarization, a process wherein the basal and apical regions of cells become distinct, leading to the first cell fate decisions. In mouse embryos, basal cells enriched in E-cadherin remain unpolarized and contribute to the inner cell mass (ICM), while apical polarized cells differentiate into the trophectoderm (TE) [26]. This differentiation results in the formation of a blastocyst comprising two distinct lineages. Furthermore, recent studies have demonstrated that the zygotic expression of Tfap2c and Tead4 is essential for initiating polarization [27].

Key genes, such as *Oct4*, *Nanog*, *Cdx2*, and *Gata6*, are pivotal in determining the fate of various lineages during early embryonic development. Oct4 and Nanog are essential for the formation of the ICM, while Cdx2 is critical for the development of the TE lineage [28]. The differentiation of the TE is regulated by the Hippo/Yes-associated protein (YAP) signaling pathway. When the Hippo pathway is inactivated, YAP translocates to the nucleus and binds to Tead4, inducing the expression of *Cdx2* [29]. This regulation contributes to the segregation of ICM and TE cell fates, starting from the morula stage [30]. In humans, monkeys, cows, and pigs, Cdx2 expression in the TE is first detected at the blastocyst stage, and TE differentiation occurs later than that in mice (Table 1) [31]. Specifically, in pig and cow blastocysts, inhibition of the extracellular signal-regulated kinase (ERK) signaling pathway at the blastocyst stage promotes TE expansion. Although data on human embryos are limited, research by Guo et al. suggests that inhibiting ERK expression in human embryonic stem cells (ESCs) similarly induces TE differentiation [32]. In monkeys, this was achieved by the combined inhibition of TGFβ/Nodal and ERK [33]. In contrast, in mice, the ERK pathway is necessary for TE formation [34]. As the blastocyst matures, the ICM differentiates into the epiblast (EPI) and the primitive endoderm (PrE). Within the ICM, cells expressing Gata6 migrate to the periphery to form the PrE [35]. Consequently, the pre-implantation embryo comprises three distinct lineages—EPI, PrE, and TE.

In mice, the FGF/ERK pathway is critical for balancing the specification of the EPI and PrE. Several studies have indicated that inhibiting the FGF/ERK pathway promotes ICM differentiation into the EPI while disrupting the formation of the PrE [31,36,37]. Specifically, the activation of the ERK pathway is essential for PrE differentiation, and its inhibition results in a considerable reduction in the number of PrE cells and an increase in that of EPI cells. However, some contradictory findings demonstrated that ERK inhibition can disrupt EPI formation [38]. Similarly, in humans, it has been reported that FGF/ERK signaling is important for PrE formation [39], in contrast to observations in a previous study [40]. In monkeys, there are reports that treating ESCs with BAM4 induces the PrE [41]. However, there is still a lack of sufficient studies in this area. In ungulates, MEK signaling is involved in the segregation of the PrE, but the fact that PrE markers are not completely suppressed when an MEK inhibitor is applied suggests the involvement of other signaling pathways in PrE formation [42,43].

In pre-implantation mouse embryos, EPI development is characterized by the high expression of Jak/Stat3 and BMP signaling components. Post-implantation, TGFβ/Nodal signaling becomes critical for further development [44]. Conversely, early EPI development in humans, monkeys [45], and pigs [46] heavily depends on TGFβ/Nodal signaling. In the bovine EPI, TGFβ/SMAD-related genes have been recently reported to be upregulated [47].

### 2.3. Differences in Gastrulation

After implantation, gastrulation is a critical developmental process that all multicellular organisms undergo to establish their basic body plan. This complex and highly regulated process begins with the formation of the primitive streak, a structure that marks the initiation of gastrulation. During this phase, cells undergo extensive movements and differentiation, forming the three primary germ layers—the ectoderm, mesoderm, and endoderm [48]. These germ layers serve as the foundational tissues from which all organs and tissues of the organism develop.

**Figure 1:**
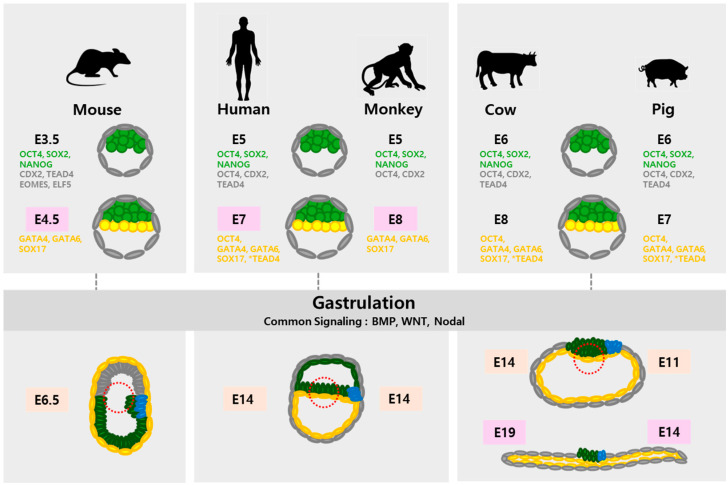
Comparison of embryonic development and the expression of lineage markers between mammalian species [35,51,54,55,58,59,60,61]. Due to the diversity of species included in the model, several species are grouped together under the general term ‘Monkey or Cow’. E, embryonic day; inner cell mass and epiblast (green); trophectoderm (grey); hypoblast (yellow); mesoderm (blue); presence or absence of bilaminar disc structures (red dotted line); implantation day (pale pink); and gastrulation day (pale orange).

As shown in Figure 1 and Table 2, there are notable differences not only in the timing of lineage separation but also in morphology among various species [45,49,50,51]. While mouse embryos develop into cup-shaped structures, embryos of humans, monkeys, cows, and pigs form flat, bilaminar disc structures. Mouse, human, and monkey blastocysts implant immediately after hatching and then initiate gastrulation. In contrast, the blastocysts of ungulates, such as cows and pigs, do not undergo implantation immediately; rather, they elongate into tubular and filamentous forms. During gastrulation, key signaling pathways involving BMP, WNT, and Activin A/Nodal orchestrate critical developmental processes [52,53,54,55]. These signals activate the posterior EPI cells, leading to the formation of the embryonic body plan, the establishment of the anterior−posterior (AP) axis, and the emergence of the primitive streak. This developmental cascade triggers the epithelial−mesenchymal transition in EPI cells, a crucial step for the subsequent differentiation into mesodermal and endodermal lineages [56,57]. 

While extensive research on mouse embryos has provided detailed insights into the process of gastrulation, our understanding of this critical developmental stage in other mammals remains relatively limited. In particular, research on human embryos is substantially constrained by bioethical considerations, which restrict investigations to the period before the formation of the primitive streak, following the “14-day rule” [62]. To overcome these limitations, recent scientific studies have focused on developing synthetic embryos through stem cell co-culture systems. These innovative synthetic models have been developed with the aim to faithfully recapitulate early embryogenesis and offer a valuable platform for studying developmental processes without ethical constraints [63]. By leveraging these advanced models, researchers can explore the intricacies of mammalian embryogenesis, extending our knowledge beyond well-characterized mouse models. Such studies hold promise for uncovering the fundamental developmental principles that are conserved across species as well as the unique aspects of human embryogenesis.

## 3. Current Status of Stem Cell Research in Cattle and Pigs

**Figure 2:**
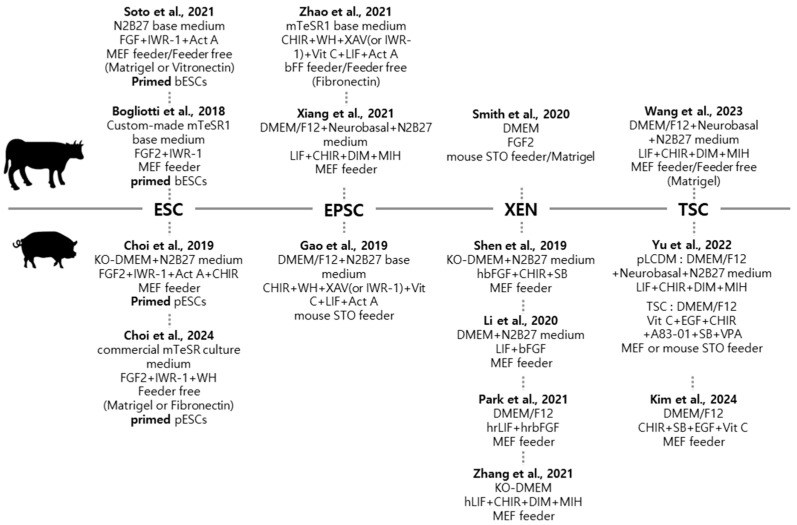
Media components used for stem cell line establishment in cows [65,66,67,68,69,70] and pigs [71,72,73,74,75,76,77,78,79]. MEF, mouse embryonic fibroblast; STO, SIM (Sandos Inbred Mice) mouse embryonic fibroblast; bFF, bovine fetal fibroblast; DMEM, Dulbecco’s Modified Eagle’s Medium; KO-DMEM, knockout Dulbecco’s Modified Eagle’s Medium; hbFGF, human bFGF; hrbFGF, human recombinant bFGF; hrLIF, human recombinant LIF; Act A, Activin A; CHIR, CHIR99021; SB, SB431542; XAV, XAV939; WH, WH-4-023; Vit C, vitamin C; DIM, (S)-(+)-dimethindene maleate; and MIH, minocycline hydrochloride.

Synthetic embryology, an innovative and rapidly evolving branch of developmental biology, delves into the intricate processes of early development through the use of “embryo-like models” called “embryoids” [64]. Leveraging the potential of stem cells and other cellular materials, researchers can circumvent the ethical and logistical challenges traditionally associated with natural embryos. These embryoids are engineered to mimic various stages of embryogenesis, spanning from the blastocyst stage to gastrulation and beyond. This approach provides unprecedented insights into the fundamental mechanisms of cell differentiation, tissue formation, and intricate signaling pathways that orchestrate early developmental processes. However, the stem cell lines required for synthetic embryology remain relatively understudied in preclinical models, particularly in large animals such as cattle and pigs. The following sections provide an overview of the current status of stem cell research in these species (Figure 2).

### 3.1. Embryonic Stem Cells (ESCs)

Embryonic stem cells (ESCs) are characterized by two distinct states of pluripotency—naïve and primed [80]. Naïve pluripotency is established in the ICM of the pre-implantation embryos and is associated with greater plasticity than that in primed pluripotency, making cells valuable tools for developmental biology and regenerative medicine research. Notably, these pluripotent states are interconvertible. For instance, naïve mouse ESCs cultured in a 2i/LIF medium can be converted into the primed state when switched to a medium containing FGF2 and WNR1 [81]. Conversely, primed human ESCs (hESCs) can be reprogrammed into the naïve state [82,83]. Recently, a formative state of pluripotency that exists between the naive and primed states has been reported, adding another layer of complexity and potential to stem cell research [84].

In ungulates, naive pluripotent cells have not yet been derived from the embryo. Unlike in mice, primed ESCs can be derived from the blastocyst stage in ungulates, similar to humans [65,71]. However, this may be due to the fact that research on protocols for culturing these cells in a naïve state remains much less focused than that on mice or humans. As a result, efforts to establish a naïve ESC are still ongoing [85,86]. In contrast, several studies have reported the successful induction and stable culturing of primed ESCs in cows [65,66] and pigs [71,72]. Particularly in cows, when the WNT inhibitor IWR1 was omitted from the culture conditions, the expression of pluripotency markers was lost, suggesting that IWR1 plays a crucial role in the successful induction and proliferation of bESCs. Subsequent studies reported the establishment of bESCs under feeder-free conditions by modifying the composition of the complex medium [66]. Interestingly, IWR1 was found to be crucial for maintaining pluripotency in feeder conditions, while Activin A was essential in feeder-free conditions. Additionally, IWR1 aids in maintaining stable long-term cultures of porcine ESCs (pESCs) in pigs [71]. Recently, pESCs have been successfully established and stably cultured under serum-free and feeder-free conditions with the combination of FGF2, IWR-1, and WH-4-023. Unlike in bESCs, Activin A cannot sufficiently sustain pluripotency under serum-free and feeder-free conditions without additional factors [72].

Since the induction of pESCs from early embryos in the 1990s, extensive research has been conducted to establish authentic pESCs [71,87,88,89]. Although attempts have been made based on the ESC culture conditions of mice and humans, authentic pESCs suitable for an in vitro culture protocol have yet to be established. This may be attributed to the limited understanding of the regulatory mechanisms involved in pig embryo development and the differences that exist across species. Although existing studies have improved our understanding of the molecular mechanisms of embryo development and pluripotency through RNA-seq [89,90,91,92], generating authentic ESCs remains an ongoing challenge.

### 3.2. Expanded Potential Stem Cells (EPSCs)

In 2019, Gao et al. [73] screened small molecules used for mEPSC, mESC, and hESC culturing and successfully induced porcine EPSCs (pEPSCs) from early pig embryos. These pEPSCs showed potential to contribute to both embryonic and extraembryonic cell lineages. The small-molecule cocktail, comprising CHIR99021, WH-4-023, XAV939, vitamin C, LIF, and Activin A, established suitable culture conditions for pEPSCs. Although synthetic embryoid research based on EPSCs has not yet been reported, the findings of Gao et al. [73] have provided valuable tools and insights for broader exploration of porcine synthetic embryoids.

In 2021, Zhao et al. established bovine EPSCs (bEPSCs) from iPSCs and blastocysts [67] by adjusting the porcine EPSC medium (pEPSCM) [73]. The researchers systematically tested various culture conditions, including those used for mESCs and hESCs, to optimize protocols for inducing and maintaining pluripotent stem cells in cows. While the original pEPSCM promoted the expression of key pluripotency genes, it was not optimal for the long-term culturing of bEPSCs. To address this limitation, the medium was adjusted, resulting in a stable protocol that supported the long-term culturing of bEPSCs. Notably, this optimized protocol allowed for the culturing of bEPSCs even in feeder-free conditions, and, similar to pEPSCs, the bEPSCs remained sensitive to the Mek1/2 inhibitor PD-0325901. In the same year, Xiang attempted to generate bEPSCs from biPSCs by using the LCDM medium (a chemical cocktail containing hLIF, CHIR99021, DiM, and MiH) previously employed for mEPSCs and hEPSCs [68]. However, while this medium successfully supported bEPSCs from iPSCs, it failed to establish bEPSCs when applied directly to blastocysts.

Additionally, recent mEPSC research [93,94] shows that, unlike EPSCs, TBLCs resemble two–four-cell-stage embryos in their transcriptomic and epigenetic profiles. They also exhibit greater bidirectional potential, contributing to both embryonic and extraembryonic tissues. If TBLCs can be established in ungulates, they could unlock new possibilities for synthetic embryoid research, offering more effective tools for modeling early embryonic development and investigating lineage decisions across species.

### 3.3. Extraembryonic Endoderm (XEN) Cells

XEN cells are a useful model for studying the PrE. In murine models, beyond their application in metabolic research [95], novel protocols have been developed to more efficiently generate XEN cells from the ICM [96]. In human studies, the signaling pathways involving WNT, Nodal, and LIF have been identified as crucial for generating cells that resemble the PrE from hESCs [97]. In contrast, research on XEN cells in cattle and pigs is relatively sparse. A recent study by Smith et al. [69] reported that treating day 8 in vitro-fertilized embryos with 10 ng/mL FGF2 and 20% fetal bovine serum and culturing them until day 15 under a Matrigel coating improved the formation efficiency of bovine XEN cells from blastocysts and enhanced their growth rate to 81.6%. However, considering the issues related to FBS usage [98], this complicates the precise understanding of XEN differentiation mechanisms. Therefore, much more work remains to be carried out on bovine XEN cells. In 2019, these cells were also derived from piPSCs [74]. The addition of Chir99021 and SB431542, along with bFGF, enhanced the pluripotency of pXENCs. From 2020 to 2021, studies reported the induction of XEN cells from porcine blastocysts [75,76,77]. However, no reports have yet confirmed the induction of XEN cells from EPSCs.

### 3.4. Trophoblast Stem Cells (TSCs)

Trophoblast stem cells (TSCs), initially isolated from the mouse placenta [99], have been the focus of extensive research aimed at understanding trophoblast development [100,101]. There is substantial evidence regarding the multipotency of TSCs and their potential to differentiate into various tissues [102]. While comparable cutting-edge research, including the differentiation of TSCs from PSCs, is emerging in human models [103,104], advancing TSC research in large animal models remains a major challenge.

Recently, Wang et al. [70] derived TSCs from bovine blastocysts by using the LCDM medium, which has been used to induce mEPSCs and hEPSCs. They confirmed that these TSCs could be maintained under feeder-free conditions and that LIF, CHIR99021, DiM, and MiH were essential for maintenance. However, considering that bovine ICM and TE have the plasticity to reform [105,106], further studies are needed to determine whether bovine TSCs are derived from the ICM or TE in the LCDM medium. In pigs, studies applying the LCDM medium conditions to embryos observed a strong tendency for differentiation into the trophoblast lineage [78]. More recently, another approach was used to successfully establish TSC lines from somatic cell nuclear-transferred blastocysts [79].

## 4. Update on the in Vitro Synthetic Embryology of Mammals

Since the development of pioneering techniques in 2014 [107,108] to culture ESCs into structures resembling early embryos, the field of synthetic embryology has witnessed remarkable advancements [5,109]. Researchers have successfully created various embryoid models, including “blastoids” and “gastruloids,” each replicating distinct developmental stages. These innovative models offer a controlled environment with which to study genetic and cellular behaviors, thereby enabling observations that would be challenging, if not impossible, in natural embryos.

In the following sections, we comprehensively review the current state of embryoid research across various species (Table 3).

### 4.1. Mice

The pioneering work of Harrison et al. [53] marked a major milestone in the field of synthetic embryology by demonstrating the creation of synthetic embryos using only ESCs and TSCs. These synthetic embryos, referred to as “ETS-embryos”, closely resemble the developmental stage of E5.5 embryos. These researchers utilized Matrigel as a 3D extracellular matrix scaffold to support the formation and growth of these structures. Subsequently, Rivron et al. [109] advanced this work by generating ETS-blastoids, which mimic E3.5 embryos, by using an agarose hydrogel microwell system under non-adherent conditions. Although these ETS-blastoids did not achieve complete embryonic development when implanted into the uterus, they exhibited a range of markers indicative of extra-embryonic tissues during the post-implantation stage. The authors identified key signaling pathways involving FGF4, interleukin (IL)-11 (a STAT regulator), BAM4, Nodal, WNT6, and WNT7B, which are crucial for trophectoderm development and successful in utero implantation. In the same year, Sozen et al. [115] reported the creation of “ETX-embryos”, which resemble embryos at the E5.5–7.5 stages, via the co-cultivation of XEN cells derived from the PrE with the ETS model in an AggreWell plate.

More recently, Dupont et al. [5] developed an advanced protocol for generating ETX embryoids, also similar to E5.5~7.5 embryos. They first created PrE-ESCs by using CRISPR-Cas9 to introduce a CAG-driven rtTA system into ESCs. The exposure of these ESCs to doxycycline induced the expression of Fgfr2 and Gata6, transforming them into PrE-like cells. ETX embryos were then generated using a time-delay technique, wherein TSCs were added 1 day after the ESCs and PrE-ESCs had aggregated in a U-bottomed 384-well plate under static culture conditions. This method demonstrated a 43% efficiency in achieving correct 3D cell distribution, significantly higher than that of the simultaneous assembly methods (7% and 30%), owing to its better potential for cell organization and development.

In addition to the use of pluripotent stem cells (PSCs) and TSCs, researchers have explored the potential of EPSCs, which possess both embryonic and extraembryonic developmental abilities [116,117]. EPSCs are derived from eight-cell-stage embryos and exhibit characteristics distinct from those of two-cell embryos [118]. Recently, stem cells with totipotent-like properties, similar to those of two-cell embryos, have been successfully derived [93,94]. Xu et al. [94] reported the in vitro induction of totipotency-like stem cells, termed totipotent potential stem cells, using a chemical cocktail. This novel approach provides new avenues for studying the regulatory mechanisms of totipotency and the formation of early pre-implantation embryos.

### 4.2. Humans and Monkeys

Recent advancements have also paved the way for the generation of gastruloids by using hEPSCs. Liu et al. [6] developed an advanced protocol to generate 3D peri-gastruloids by cultivating hEPSCs in AggreWell plates with a specialized medium. These peri-gastruloids, although incomplete due to the lack of trophoblasts, can be cultured for approximately 11 to 13 days. During this period, they exhibit developmental characteristics similar to those of cynomolgus monkey embryos cultured for 23 to 25 days and in vivo embryos at day 20. Additionally, protocols that successfully replicate trophoblast formation have been reported. Weatherbee et al. [114] demonstrated the generation of 3D gastruloids by co-culturing wild-type hESCs with hESCs induced to overexpress extraembryonic factors using the Tet-On system. Despite these groundbreaking advancements, the efficiency of model generation remains relatively low.

In non-human primates, blastoid studies have been exclusively reported in cynomolgus monkeys (*Macaca fascicularis*). Li et al. [60] successfully generated monkey blastoids, which resemble E8-9 embryos, using naive cynomolgus embryonic stem cells. They developed a two-step induction protocol by modifying the existing human blastoid generation method. These blastoids can be stably cultured in vitro for approximately 17 days. When implanted into surrogate monkeys, early signs of pregnancy were observed, although further development was limited. This highlights the need to optimize culture conditions, as the efficiency of model generation is still low, and TE differentiation may be immature.

### 4.3. Cows and Pigs

Pigs are often used as models for cross-species organ transplantation and preclinical large-animal studies owing to their anatomical and physiological similarities to humans [119]. Similarly, cows exhibit greater similarity to humans in early embryonic development than do mice, as evidenced by transcriptome comparisons [120]. This suggests that cows could serve as a model for assessing the impact of assisted reproductive technologies on humans. However, research on embryo development using stem cells in these species remains limited.

In cows and pigs, blastoid studies have been reported with promising results. Pinzón-Arteaga et al. [112] developed an efficient protocol for generating 3D blastoids by co-culturing bovine EPSCs and TSCs. They modified the FAC (FGF2, Activin-A, and CHIR99021) medium, initially used for differentiating hypoblast-like cells from naïve hPSCs, to suit bovine blastocyst formation. To enhance preimplantation embryo development in the bovine model, they added LIF, reduced the concentration of FGF2, and introduced PD, an MEK inhibitor, to regulate the fates of the ICM and hypoblast. This optimized medium conditions (tFACL+PD) and helped achieve a high-efficiency blastoid generation rate of 64.2 ± 7.6%. These blastoids can be cultured for more than 2 weeks and can induce the physiological signals necessary for maintaining pregnancy in surrogates to an extent similar to that achieved with in vitro fertilization blastocysts. However, further research is needed to improve the accuracy of comparisons and better assess the developmental potential of bovine blastoids due to limitations, such as differences between bovine blastoids and blastocysts, potential biases from different single-cell RNA-sequencing platforms, and a lack of late-stage blastocyst data.

Xiang et al. [113] reported a method for deriving ESCs, using a novel culture medium (4FIXY medium) from parthenogenetic embryos and generating blastoids through a two-step differentiation strategy using 4FXY (without IWR1) and the iBlastoid medium in a 3D culture system. These blastoids closely resembled porcine blastocysts in terms of morphology, size, cell lineage composition, and single-cell transcriptome characteristics. They also survived and expanded for 18 days under two different culture conditions via prolonged in vitro culturing. This result provides a valuable foundation for future research aimed at optimizing in vitro culture conditions and improving the quality and developmental potential of porcine blastoids.

## 5. Future Directions and Conclusions

This review examined the advancements in the research on the current synthetic embryo model, specifically embryoids, with a focus on both conserved and species-specific mechanisms of embryonic development across various species, including rodents, humans, non-human primates, and ungulates. The advent of 3D culture technologies and the development of synthetic embryo models have unlocked unprecedented opportunities with which to study early embryogenesis in vitro. These innovative models have enabled a profound comprehension of the complex processes involved in mammalian embryonic development, including key events, such as gastrulation and neurulation, without the ethical and logistical constraints inherent in in vivo studies. Despite these advancements, further research is imperative to elucidate the mechanisms governing stem cell lineages across different species. A critical step in this direction involves the integration of cutting-edge technologies, such as single-cell transcriptomics [90] and gene editing [5,114]. These tools will facilitate the systematic and comprehensive analysis of the molecular and cellular mechanisms underpinning embryogenesis. The insights obtained from such in-depth research will not only enhance the development of systems and platforms for establishing desired stem cell lines and improving differentiation efficiency, but also provide invaluable resources for bridging the knowledge gaps in embryonic development by identifying species-specific variations. As the field of synthetic embryology continues to evolve, it stands at a critical juncture. Future research is poised to further refine these models, thereby considerably advancing our understanding of embryogenesis. Such advancements can potentially pave the way for the development of innovative therapeutic strategies in regenerative medicine, marking the beginning of a new era in medical science and biotechnology.

## Figures and Tables

**Table 1:** Comparison of species-specific mechanisms in compaction and cell fate decisions.

Species	Mouse	Human	Monkey	Cow	Pig
Compaction cell stage	8	4–16	16–32	16–32	16–32
Common major molecules in compaction	E-cadherin (mediating cell–cell adhesion)				
Key signaling for EPI development	Jak/Stat3, BMP	TGFβ/Nodal	TGFβ/Nodal	TGFβ/SMADs	TGFβ/Nodal
Key signaling for PrE development	FGF/ERK	FGF/ERK	BMP4	FGF/ERK, other	FGF/ERK, other
ERK pathway in first TE fate	Promoting the TE fate	Upon inhibitor treatment, lack data in embryo, naïve hESC into TE	Lack data in embryo; upon TGFβ/Nodal and ERK inhibitor treatment, can obtain TSCs	Upon inhibitor treatment, expansion of the TE	Upon inhibitor treatment, expansion of the TE

**Table 2:** Comparison of gastrulation across mammalian species.

Species	Mouse	Human	Monkey	Cow	Pig
Initiation	E6.25-	E14-	E14-	E14-	E11-
Signaling	BMP, WNT, and Nodal				
Morphology	Cup shape	Flat, bilaminar disc structures		Flat, bilaminar disc structures that then elongate into tubular and filamentous forms	

**Table 3:** Summary of blastoid and gastruloid culture in mammals since 2022.

A. Blastoids							
Species	Cell Source	Platform	Seeding	Basal Media	Media Supplementation	Efficiency	References
Human	Naïve hESCs hiPSCs	24-well AggreWell 400	H9, YN-hESCs: 30,000 cells/well WIBR3-hESCs, RC-hiPSCs, and YN-hiPSCs: 36,000 cells/well Other cell lines: optimization of the initial cell number is required	eHDM: DMEM/F12:neurobasal (1:1), N2B27 eTDM: DMEM/F12:neurobasal (3:1), N2B27	eHDM: bFGF, Activin A, Chir99021, CEPT cocktail eTDM: KSR, PD0325901, A83-01, SB590885, WH-4-023, rhLIF, LPA, and CEPT cocktail	_	[110]
	Primed hESCs convert to naïve	24-well AggreWell 400	100,000 cells/well	N2B27+NaPy and BIM: DMEM/F12:neurobasal (1:1), N2B27	N2B27+NaPy BIM: NaPy, ITS-X, KOSR, PD0325901, A83-01,WH-4-023, IM-12, rhEGF, ascorbic acid, and valproic acid	Blastoid cavitation up to 90%	[7]
	Naïve hESCs hiPSCs	96-well plate (non-adherent hydrogel microwells)	3.0 × 10^4^ cells/well	PXGL and PALLY: DMEM/F12, neurobasal (1:1), N2B27	PXGL media: BSA, PD0325901, XAV-939, Gö 6983, and hLIF PALLY media: BSA, PD0325901, A 83-01, LPA, hLIF, and Y-27632	More than 70%	[111]
Cynomolgus monkey	Naïve cyESCs	24-well AggreWell 400	30,000 naïve cyESCs/well	HDM and mTDM: DMEM/F12, neurobasal (1:1), N2B27	HDM: bFGF, Activin A, and CHIR99021 mTDM: ITS-X, Na-pyruvate, KSR, BSA, PD0325901, A83-01, CHIR99021, SB431542, IWR-1, TSA, DZNep, rhLIF, human Activin A, EGF, L-ascorbic acid, and VPA	25%	[60]
Cattle	bEPSC bTSC	24-well AggreWell 400	19,200 bEPSCs/well 19,200 bTSCs/well	DMEM/F12:neurobasal (1:1), N2B27	BSA, ITS-X, LIF, Activin A, FGF2, PD032590, Chir99021, and CEPT cocktail	64.2% ± 7.6%	[112]
Pig	pESCs	Ultra-low attachment multiple-well plates	40,000 pESCs/well	4FXY and iBlastoid: DMEM/F12:neurobasal (1:1), N2B27	4FXY: iBlastoid minus hLIF, BMP4, bFGF, SB431542, DZNep, and TSA iBlastoid: KOSR, 2-Phospho-l-ascorbic acid trisodium salt, human IL-6, human sIL-6 Receptor α, Activin A, hLIF, human IGF1, BMP4, bFGF, CHIR99021, XAV939, SB431542, Y-27632, DZNep, and TSA	29.68%	[113]
**B. Gastruloids**							
**Species**	**Cell Source**	**Platform**	**Seeding**	**Basal Media**	**Media Supplementation**	**Efficiency**	**References**
Mouse	mESCs PrE-ES (Ffgr2-E2A-Gata6)mTSCs	AggreWell 800 or 384-well low adhesion plate	well: (microwell or well) mESCs: 20~24 cells/well PrE-ES: 5~10 cells/well mTSCs: 70 cells/well	ETX: DMEM IVC1: Advanced DMEM/F-12	Method B ETX: 15% FBS, sodium pyruvate, and doxycycline IVC 1: 20% FBS, ITS-X, β-oestradiol, progesterone, and N-acetyl-l-cysteine IVC 2: IVC1 with 30% FBS instead of 20% FBS	40%	[5]
Human	Primed hESCs and hiPSCs convert into hEPSCs	(1) 24-well AggreWell 400 (2) 24-well AggreWell 800	Method 1 (1)5.4~10.8 × 10^4^ cells/well (2) 1.35~2.7 × 10^4^ cells/well Method 2 (2) 1.35~2.7 × 10^4^	tHDM: DMEM/F12:neurobasal (1:1), N2B27 IVC1, 2: Advanced DMEM/F12	tHDM: FGF2, Activin-A, CHIR99021, PD0325901, and CEPT cocktail tHDM(-CEPT cocktail) tHDM(-Chir) IVC1: ITS-X, β-estradiol, progesterone, N-acetyl-l-cysteine, sodium pyruvate, 5% FBS, and 4% Matrigel IVC2: IVC1 with 30% KSR instead of 20% FBS IVC2 plus D-glucose	26.67%	[6]
	Primed hESCs (PrE using Tet-On system; GATA6 or SOX17 or GATA3 or TFAP2C)	24-well AggreWell 400 and ultra-low attachment 96-well plates	Wild-type ESCs and hypoblast-like cells: each with 9600 cells/well Trophoblast-like cells: 19,200 cells/well	N2B27: DMEM/F12:neurobasal A (1:1), N2B27 hIVC1: Advanced DMEM/F12	N2B27: doxycycline, KSR hIVC1: 20% FBS, hIGF1, ITS-X, glucose, sodium lactate, β-estradiol, and progesterone	-	[114]

“Media supplementation” indicates the additives used in the culture of synthetic embryoids. Abbreviations: CEPT cocktail, Chroman 1, Emricasan, polyamine supplement, and TransISRIB; rhLIF, recombinant human LIF; hLIF, human LIF; BSA, bovine serum albumin; LPA, 1-oleoyl lysophosphatidic acid sodium salt; ITS-X, Insulin-Transferrin-Selenium-Ethanolamine; NaPy, sodium pyruvate; and KOSR or KSR, knockout serum replacement.

## Abbreviations

3D	three-dimensional
EGA	embryonic genome activation
ERK	extracellular signal-regulated kinase
ICM	inner cell mass
Epi	epiblast
PrE	primitive endoderm
TE	trophectoderm
PSC,	pluripotent stem cell
EPSC	expanded potential stem cells
ESC	embryonic stem cell
hESCs	human ESCs
bESCs	bovine ESCs
pESCs	porcine ESCs
EPSCs	expanded potential stem cells
bEPSCs	bovine EPSCs
pEPSCM	porcine EPSC medium
pEPSCs	porcine EPSCs
hEPSCs	human EPSCs
XEN cell	extraembryonic endoderm (XEN) cell
TSC	trophoblast stem cell
MEF	mouse embryonic fibroblast
STO	SIM (Sandos Inbred Mice) mouse embryonic fibroblast
bFF	bovine fetal fibroblast
DMEM	Dulbecco’s Modified Eagle’s Medium
KO-DMEM	knockout Dulbecco’s Modified Eagle’s Medium
hbFGF	human bFGF
hrbFGF	human recombinant bFGF
hrLIF	human recombinant LIF
Act A	Activin A
CHIR	CHIR99021
SB	SB431542
XAV	XAV939
WH	WH-4-023
Vit C	vitamin C
DIM	(S)-(+)-dimethindene maleate
MIH	minocycline hydrochloride
CEPT cocktail	Chroman, Emricasan, polyamine supplement and TransISRIB
rhLIF	recombinant human LIF
hLIF	human LIF
BSA	bovine serum albumin solution
LPA	1-oleoyl lysophosphatidic acid sodium salt
ITS-X	Insulin-Transferrin-Selenium-Ethanolamine
NaPy	sodium pyruvate
KOSR or KSR	knockout serum replacement
YAP	Yes-associated protein
AP	anterior–posterior
